# ﻿Spatial decoupling of taxon richness, phylogenetic diversity and threat status in the megagenus *Erica* (Ericaceae)

**DOI:** 10.3897/phytokeys.244.124565

**Published:** 2024-07-10

**Authors:** Michael D. Pirie, Dirk U. Bellstedt, Roderick W. Bouman, Jaime Fagúndez, Berit Gehrke, Martha Kandziora, Nicholas C. Le Maitre, Seth D. Musker, Ethan Newman, Nicolai M. Nürk, E. G. H. Oliver, Sebastian Pipins, Timotheus van der Niet, Félix Forest

**Affiliations:** 1 University Museum, University of Bergen, Postboks 7800, N-5020 Bergen, Norway; 2 Department of Biochemistry, University of Stellenbosch, Private Bag X1, Matieland 7602, South Africa; 3 Hortus botanicus Leiden, Leiden University, P.O. Box 9500, 2300 RA, Leiden, Netherlands; 4 Naturalis Biodiversity Center, P.O. Box 9517, 2300 RA, Leiden, Netherlands; 5 Institute of Biology Leiden, Leiden University, PO Box 9505, 2300 RA Leiden, Netherlands; 6 Universidade da Coruña, BIOCOST research group, Centro Interdisciplinar de Química e Bioloxía (CICA), Rúa As Carballeiras, 15071, A Coruña, Spain; 7 Universidade da Coruña, Departamento de Bioloxía, Facultade de Ciencias, 15071, A Coruña, Spain; 8 Department of Botany, Faculty of Science, Charles University, 128 00 Prague, Czech Republic; 9 Senckenberg Biodiversity and Climate Research Centre, Senckenberg Gesellschaft für Naturforschung, 60325 Frankfurt am Main, Germany; 10 Department of Genetics, University of Stellenbosch, Private Bag X1, Matieland 7602, South Africa; 11 Department of Biological Sciences, University of Cape Town, Private Bag, Rondebosch 7701, South Africa; 12 Centre for Functional Biodiversity, School of Life Sciences, University of KwaZulu-Natal, Scottsville, Pietermaritzburg 3209, South Africa; 13 Department of Plant Systematics, Bayreuth Centre of Ecology and Environmental Research (BayCEER), University of Bayreuth, Universitätsstraße 30, 95447 Bayreuth, Germany; 14 Department of Botany and Zoology, University of Stellenbosch, Private Bag X1, Matieland 7602, South Africa; 15 Royal Botanic Gardens, Kew, Richmond, TW9 3AE, UK; 16 Department of Life Sciences, Imperial College, London, UK; 17 On the Edge, London, UK

**Keywords:** Conservation prioritisation, heathers, large genera, phylogeny, threatened species

## Abstract

Estimates of the number of vascular plant species currently under threat of extinction are shockingly high, with the highest extinction rates reported for narrow-range, woody plants, especially in biodiversity hotspots with Mediterranean and tropical climates. The large genus *Erica* is a prime example, as a large proportion of its 851 species, all shrubs or small trees, are endemic to the Cape Floristic Region (CFR) of South Africa. Almost two hundred are known to be threatened and a further hundred are ‘Data Deficient’. We need to target conservation efforts and research to fill the most problematic knowledge gaps. This can be especially challenging in large genera, such as *Erica*, with numerous threatened species that are closely related. One approach involves combining knowledge of phylogenetic diversity with that of IUCN threat status to identify the most Evolutionarily Distinct and Globally Endangered (EDGE) species. We present an expanded and improved phylogenetic hypothesis for *Erica* (representing 65% of described species diversity) and combine this with available threat and distribution data to identify species and geographic areas that could be targeted for conservation effort to maximise preservation of phylogenetic diversity (PD). The resulting 39 EDGE taxa include 35 from the CFR. A further 32 high PD, data deficient taxa are mostly from outside the CFR, reflecting the low proportion of assessed taxa outside South Africa. The most taxon-rich areas are found in the south-western CFR. They are not the most phylogenetically diverse, but do include the most threatened PD. These results can be cross-referenced to existing living and seed-banked *ex situ* collections and used to target new and updated threat assessments and conservation action.

## ﻿Introduction

The world’s biosphere is currently experiencing a human-mediated mass extinction (Lughadha et al. 2020), with habitat destruction and degradation, pollution, invasive alien species and climate change extirpating species (IPBES 2019). These processes are dramatically reducing numbers and genetic diversity of populations and impacting the viability of their complex interdependencies with other organisms (Pollock et al. 2020). Over a third of vascular plant species are estimated to be under threat of extinction (e.g. 39%, Lughadha et al. (2020); 45%, Bachman et al. (2023)). The highest extinction rates are reported for narrow-range, woody plants, particularly those in Mediterranean climate and tropical biodiversity hotspots (Humphreys et al. 2019).

The genus *Erica* (of the heather family, Ericaceae) is a prime example of such a group of plants. One of the largest flowering plant genera (Frodin 2004), its 851 species (Elliot et al. 2024) are all woody. They are distributed from Europe to southern Africa, with significant diversity at higher elevations across tropical Africa and Madagascar, but concentrated in the Mediterranean-type climate of the Cape Floristic Region (CFR) of South Africa, a world biodiversity hotspot (Myers et al. 2000). Within a modest geographical extent (ca. 90,000 km^2^), the CFR is home to a disproportionately high number of plant species (> 9,000), most of which are found nowhere else (70% species endemism) (Linder 2003). Of this spectacular and unique flora, around 7% of the species richness is represented by over 700 species of *Erica*. These are abundant in many CFR communities, mostly found in fynbos habitats which are subject to regular fires after which they are adapted either to re-seed or to resprout (Ojeda 1998; Segarra-Moragues and Ojeda 2010). Individually, the species often exhibit patterns of narrow local endemism (Oliver et al. 1983).

Habitat destruction and degradation have already resulted in species extinctions in *Erica* and, due to their restricted ranges, many are endangered. The South African National Biodiversity Institute (SANBI)’s Red List includes 944 taxa of *Erica* for South Africa (species, subspecies and varieties) of which 108 are classified as rare, a further 84 as vulnerable (VU), 60 endangered (EN) and 46 critically endangered (CR). Three are already extinct in the wild (EW) (Raimondo et al. 2009). Furthermore, over a hundred species are classified as ‘Data Deficient’, their populations insufficiently known to allow us to estimate the degree of threat they face. Such taxa are more likely to be rare and threatened too (Bachman et al. 2023).

Resources for conservation are limited and efforts need to focus on meaningful priorities. For example, the most critically-endangered species might be prioritised as an immediate response to prevent extinction and those not already protected in *ex situ* collections might be targeted for seed banking or cultivation in botanic gardens (Westwood et al. 2021). In *Erica*, two species have been saved from the brink of extinction by a combination of the fortuitous preservation of living collections and concerted action to re-introduce them into the wild: *Ericaverticillata* P.J.Bergius (Hitchcock & Rebelo, 2017) and *E.turgida* Salisb. Substantial efforts have been made to preserve material in seed banks of other threatened species before their last wild populations are lost (Liu et al. 2020). Ideally, species would be conserved in their native habitats, i.e. *in situ*, as parts of species assemblages that may include further threatened taxa. With numerous threatened taxa distributed across a complex mosaic of habitats, we need formal criteria to decide which species and which areas should have priority.

Potential criteria for conservation prioritisation include threat status of individual species and numbers of such species in given areas. However, species are not equal in evolutionary terms. Extinction destroys unique lines of evolutionary innovation by removing branches from the tree of life. The long branch of an isolated species on the tree of life represents more unique evolutionary history, or ‘phylogenetic diversity’ (PD) (Faith 1992), than the short branch of a recently-evolved species with several extant close relatives. PD, a metric compiled from the sum of all the branches linking a set of species on a phylogenetic tree, can be used in combination with threat status to derive phylogenetically informed conservation priorities, such as through the Evolutionarily Distinct and Globally Endangered (EDGE) approach (Isaac et al. 2007). A prioritisation approach that takes PD into account could deliver very different results in a group such as *Erica*. South Africa is the most species rich area for *Erica* species, with a well-established centre of diversity within the Western Cape (Oliver et al. 1983) including many of the known threatened taxa (Raimondo et al. 2008). However, CFR diversity appears to be represented exclusively by a single Cape clade that shares a relatively recent common ancestor (Pirie et al. 2016). The geographic distribution of threatened phylogenetic diversity may not reflect that of threatened species or of species richness overall.

To estimate the evolutionary distinctiveness of each *Erica* species in a geographical framework, we need a robust phylogenetic hypothesis representing as many species of the genus as possible. The most comprehensive molecular phylogenetic tree of *Erica* currently available is that of Pirie et al. (2016) who included ca. 60% of species from across the distribution of the genus and based on DNA sequence data from the plastid genome (cpDNA) and nuclear ribosomal gene region (nrDNA). An exemplar sampling approach of multiple plastid markers delivered increased support particularly for deeper nodes (Pirie et al. 2016) and within the limits of phylogenetic resolution, the trees based on plastid and nrDNA data were largely congruent. Going forward, we need: a) to reduce the current 40% shortfall of species, b) improved resolution of the nrDNA tree to better test the degree to which cpDNA might track the *Erica* species tree and c) to reduce the substantial remaining phylogenetic uncertainty, particularly within the large Cape clade.

In this paper, we develop an expanded and improved phylogenetic hypothesis for *Erica*. Using the phylogeny, we analyse extensive openly available threat and distribution data to summarise both the taxa and areas that harbour most phylogenetic diversity, and whether that diversity is known to be, or could be threatened with extinction. These results can be cross-referenced to existing living and seed-banked ex situ collections and used to help target new and updated threat assessments and to prioritise conservation action.

## ﻿Materials and methods

### ﻿Taxon and molecular sampling

We generated new data from 81 new field-collected, silica-dried leaf samples and additional data from 79 previously analysed samples, expanding existing datasets to include a total of 730 accessions representing 551 *Erica* species (587 specific and subspecific taxa) and six outgroup taxa (four species). This represents 65% of 851 currently recognised (non-hybrid) species (Elliot et al. 2024) following the taxonomic concepts of E.G.H. Oliver (Oliver et al. 2024). In summarising known threat status and taxonomic data for use in the EDGE analyses (see below), we compiled an extended list of 1048 species, subspecies and varieties (Suppl. material 1). This number included a proportion of subspecific taxa which are validly described and for which threat status may have been formally assessed, but which may be of questionable taxonomic status. Of this more inclusive list, 55% were represented in the phylogenetic analyses. Accession details are presented in Suppl. material 2 (table; https://doi.org/10.15468/tae99n) and Suppl. material 3 (a Google Earth map). The existing body of published sequence data comprises broad taxon sampling of the plastid (cpDNA) *trnT-trnL-trnF-ndhJ* region (including genes and intervening introns and spacers) and of the nuclear ribosomal (nrDNA) internal transcribed spacer (ITS) region (including partial flanking 18S and 26S genes) and sparser sampling of cpDNA*atpI-atpH* spacer, *trnK-matK* intron and *matK* gene, *psbM-trnH* spacer, *rbcL* gene, *rpl16* intron, *trnL-rpl32* spacer and part of the nrDNA external transcribed spacer (ETS). To incorporate our new samples, we sequenced the two best represented cpDNA and nrDNA markers for *Erica*, i.e. parts of *trnT-trnL-trnF-ndhJ* and ITS and, to improve support for relationships in the nrDNA tree, we extended our sampling approach to include ETS for a subset of taxa (including some of the same samples used in Pirie et al. (2016)).

### ﻿Lab protocols

We used two different lab protocols for Sanger sequencing: 1) Direct amplification (without DNA isolation) using the method of Bellstedt et al. (2010); and 2) DNA isolation, (followed by separate PCR) using the DNeasy Plant Mini Kit (Qiagen, Hilden, Germany). In both cases, leaf material was ground using a Qiagen Tissuelyser (Retsch GmbH & Co., Haan, Germany).

PCR primers and protocols followed Mugrabi de Kuppler et al. (2015) and Pirie et al. (2017) (for ETS). We included per 25 μl reaction 2.5 μl 10× buffer, 2.0 μl 25 mM MgCl_2_, 1.0 μl 5 mM dNTPs, 0.25 μl 4 μg/μl BSA, 1 μl DMSO (ITS only), 0.1 μl Taq polymerase, 0.25 μl each of 20 μM solutions of the two primers and 1 μl DNA template. For PCR clean-up before sequencing, PCR products were treated in the original PCR reaction tube by addition of a 10 μl solution including 0.025 of 20 units/μl exonuclease I (Fermentas Life Sciences), 0.25 μl of 1 unit/μl shrimp alkaline phosphatase (Promega) and incubation (in a thermocycler) at 37 °C for 30 min and at 95 °C for 5 min. One μl of the resulting product was used for cycle-sequencing with the primers reported by Mugrabi de Kuppler et al. (2015) and Pirie et al. (2017) using Applied Biosystems (Foster City, CA, USA) Big Dye terminator kits according to the manufacturer’s instructions. Cycle-sequencing products were analysed using an automatic sequencer 3130XL Genetic Analyzer (Applied Biosystems).

### ﻿Alignment, data assessment and phylogenetic inference

We aligned new sequences to alignments of Pirie et al. (2016) by eye in Mesquite (Maddison and Maddison 2021), adopting those of the *atpI-atpH* spacer, *trnK-matK* intron and *matK* gene, *psbM-trnH* spacer, *rbcL* gene, *rpl16* intron and *trnL-rpl32* spacer without change. We performed preliminary phylogenetic analyses of markers separately under Maximum Likelihood (ML) as implemented in RAxML (as below), to identify any topological differences within plastid and nrDNA datasets that would indicate experimental error or paralogy. Individual markers were imported into SequenceMatrix (Vaidya et al. 2011) which was used to export concatenated matrices (nrDNA, cpDNA and all markers) for further analyses.

To infer topologies and clade support for cpDNA and nrDNA gene trees, we analysed matrices under ML. Analyses were performed in RAxML v. 8.1.22 (Stamatakis 2014) under the GTR-CAT model and including 1000 rapid bootstrap analysis with bootstrap support (BS) presented on the best scoring ML tree. We assessed conflict between nrDNA and cpDNA gene trees by visual inspection, comparing nodes subject to 70% or higher bootstrap support. Where we identified gene tree conflict, prior to combined analysis, the taxa with conflicting phylogenetic signals were divided into separate cpDNA and nrDNA taxa, each to be represented by one gene tree partition only. The latter allowed us to include taxa showing evidence for reticulate processes or incomplete lineage sorting in downstream analyses without violating the assumption of a strictly bifurcating tree (Pirie et al. 2009). To obtain ultrametric phylogenetic trees reflecting phylogenetic uncertainty, we performed rate smoothing on the best ML tree and 100 randomly-selected trees from the bootstrap analysis using the Penalized Likelihood (PL) approach as implemented in the function chronos in the R package ape v.5.7 (Paradis and Schliep 2019; R Core Team 2022). Before analysis, we removed outgroup taxa and tested different assumptions for among-branch-substitution-rate variation in transforming branch lengths on the ML tree in order to approximate the divergence time estimates in Pirie et al. (2016). In the final analysis on the ML and the 100 bootstrap trees, one rate category reflecting a strict clock model was optimised for 200 iterations per tree using a rate smoothing parameter of 1 and calibrated using a secondary calibration point derived from a wider fossil-calibrated analysis of Ericaceae (Schwery et al. 2015), also following Pirie et al. (2016); (crown node of Ericeae - *Erica*, *Calluna* and *Daboecia* - constrained at 62 Ma).

### ﻿Species distributions

We used geo-referenced distribution data obtained by a GBIF-query searching for “*Erica*” (11.05.2023, GBIF.org 2023) which delivered 801,625 records. We removed occurrences outside the native range of the genus and then processed the data using the “CoordinateCleaner v. 2.0-20” R package (for details see: “GBIF_occurence_cleaning_Erica_2023-05-16.R”), filtering by CoordinateCleaner::clean_coordinates with tests = c(“capitals”, “centroids”, “equal”, “gbif”, “institutions”, “seas”, “zeros”). We retained many records from South Africa represented by centroids of quarter degree squares (QDS, equivalent to a grid of ca. 25 km × 27 km) which matched the precision of additional distribution data available from the Genus *Erica* Interactive Identification Key (Oliver et al. 2024). We renamed records as necessary, based on accepted names and synonymy derived from WFO (Elliot et al. 2024) as of May 2023 (Suppl. material 4). Combination resulted in a global dataset of *Erica* with 659,696 occurrence records. A summary of numbers of records per taxon and a presence/absence matrix for taxa per QDS across the total distribution of the genus is presented in Suppl. material 5.

### ﻿EDGE priority list

We used the Evolutionarily Distinct and Globally Endangered (EDGE) approach as described in Gumbs et al. (2023). Though EDGE is typically calculated across large clades of species related at the class-level, it can be applied to smaller monophyletic groups where there is interest in maintaining a group’s phylogenetic diversity. We therefore used the approach with the set of 100 dated phylogenetic trees (see above) and the most recent conservation assessments for *Erica* species (Raimondo et al. 2009) to produce an *Erica*-specific EDGE species priority list. Given that the approach requires a complete species level tree, species for which DNA sequence data were not available and, thus, were missing in the tree, were added to the tree using the function addTaxa from the R package addTaxa (Mast et al. 2015; https://github.com/eliotmiller/addTaxa), which binds the missing species to a randomly-selected close relative. Here, we assigned the European species to one of five lineages, based on current and previous results (Mugrabi de Kuppler et al. 2015), while, within the single African clade comprising the rest of the diversity of the genus, we assigned species to clades following the strong geographic patterns uncovered by Pirie et al. (2019). All Cape species sampled to date in phylogenetic analyses are found within a single clade comprising exclusively Cape species. We assumed that all unsampled Cape species will be assigned to this Cape clade. The other African and Madagascan species belong to an “Afrotemperate” clade, with the exceptions of *E.arborea* L. (widespread, but grouped with the European *E.lusitanica* Rudolph in the “ARB” clade), the subspecies of *E.trimera* (Engl.) Beentje (“TRIM” clade) and the subspecies of *E.kingaensis* Engl. (“KIN” clade). The imputation step was replicated on all 100 ultrametric trees to take into account the phylogenetic uncertainty associated with both the reconstruction process and the imputation of missing species.

We computed EDGE scores for all species of *Erica* using the EDGE2 protocol (Gumbs et al. 2023), once for each of the 100 dated complete species-level trees (i.e. including imputed missing species). We took into account uncertainty in the probability of extinction by sampling a distribution of extinction probability, based on the Red List category of a species (see Gumbs et al. (2023) for details). Extinct species are assigned a probability of extinction of 1.0 and extinction probabilities are sampled across the distribution for DD and NE species. Of the 1,048 taxa recognised here (combining assessments from Raimondo et al. (2009) and IUCN (2023)), 51 are Critically Endangered (CR), 62 are Endangered (EN), 86 are Vulnerable (VU), nine are Near Threatened (NT), 562 are Least Concerned (LC), four are Extinct (EX) and 274 are either Data Deficient (DD) or Not Evaluated (NE). These analyses result in 100 EDGE scores for each species, obtained from the 100 trees. A species is considered an EDGE species if it is both threatened and has an EDGE score above the median EDGE score for all species in at least 95% of the iterations (i.e. trees; Gumbs et al. (2023)). We also produced a list of species that have an EDGE score above the median in 95% of the iterations, but which are either DD or NE; this list is referred to as the EDGE Research list by Gumbs et al. (2023).

We also explored spatial phylogenetic patterns of species richness and phylogenetic diversity. We compiled taxon richness and EDGE taxon richness values for each quarter degree square (QDS) where *Erica* species are found. In addition, we also calculated the phylogenetic diversity (Faith 1992) and the expected PD loss for each QDS. Phylogenetic diversity is the sum of all branches linking a set of terminals on a phylogenetic tree, while expected PD loss is the amount of evolutionary diversity that is at risk of extinction given the probability of extinction associated with each terminal. Phylogenetic diversity was calculated for each QDS by pruning the dated trees (i.e. set of 100 dated trees used for species prioritisation; see above) so that they were reduced to only the terminals found within a given QDS. PD was then compiled by summing the branch length of the pruned trees. The same approach was used to compile expected PD loss, but this time using the extinction-risk weighted trees produced by the EDGE score compilation (Gumbs et al. 2023). Median values from the 100 trees were compiled and mapped.

## ﻿Results

### ﻿DNA sequencing and alignment

Alignment of DNA sequences was generally unambiguous, except for patterns of length variation in the *trnT-L* spacer for which several positions of the alignment were problematic and excluded from analyses (1–27, 111–150, 212–224, 342–665, 672–877, 984–1012, 1097–1107, 1150–1182, 1279–1360, 1462–1491, 2031–2049, 2139–2155, 2399–2437); three shorter regions in ETS (1–15, 784–811, 1023–1178) were also excluded.

For four taxa (E.banksiivar.banksii EO12873, *E.caffra* MP655, *E.filago* BG68 and *E.insignis* [= *E.adelopetala*] MP1290), we failed to obtain plastid data, but chose to include them in the analyses, based on nrDNA only. nrDNA sequences of a small number of taxa consistently showed polymorphism indicating multiple copies were present and the resulting consensus would incorporate paralogy (*Ericaarticularis* L., E.glabellaThunb.ssp.glabella, *E.longipedunculata* G.Lodd., E.macowaniissp.lanceolata (Bolus) E.G.H.Oliv. & I.M.Oliv., E.paucifoliassp.squarrosa (Benth.) E.G.H.Oliv., *E.petraea* Benth., *E.schlechteri* Bolus, *E.seriphiifolia* Salisb., *E.syngenesia* Compton, *E.tenuifolia* L., E.venustifloraE.G.H.Oliv.ssp.venustiflora and *E.viscosissima* E.G.H.Oliv.). These were excluded. Matrices of concatenated cpDNA and nrDNA represented 726 and 730 accessions, respectively. Sequence matrices are presented in Suppl. material 6.

### ﻿Phylogenetic tree inference

**Figure 1.**
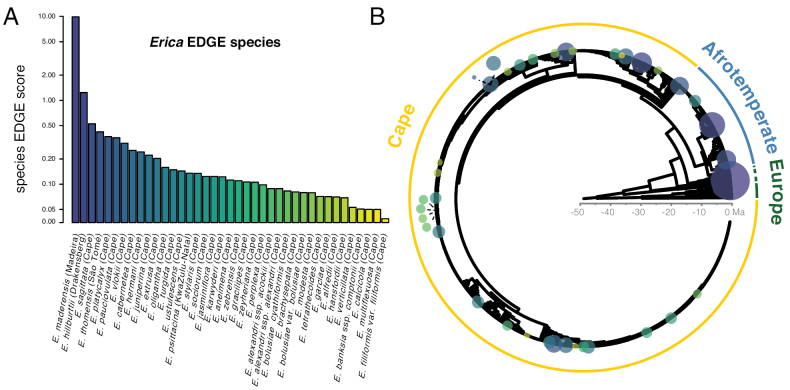
*Erica*EDGE species ranked by EDGE score (**A**) and indicated on the *Erica* phylogenetic tree (**B**; tree 69 of the 100 complete species level trees with missing taxa imputed) by circles coloured and size-scaled according to species EDGE scores. Scores are given in natural logarithmic scale..

Analyses of individual cpDNA markers showed no supported topological conflicts, so we concatenated the data in a single cpDNA supermatrix. The two nrDNA markers also showed consistent results. The resulting cpDNA and nrDNA phylogenetic trees are presented in Suppl. material 7 and all data are archived at TreeBase (study accession URL: http://purl.org/phylo/treebase/phylows/study/TB2:S30617). By comparing cpDNA and nrDNA gene trees, we identified 22 taxa with conflicting positions with bootstrap support ≥ 70%, including four that represented common patterns of conflict shared by different accessions of the same taxon (3.4% of the taxa analysed). One involved the European species *E.lusitanica* (both accessions f_ANA and PJ) that is sister to European clades EUR4/EUR5 (cpDNA) and to *E.arborea* (ARB; nrDNA). One accession of *E.woodii* Bolus (RC513) and one of *E.flanaganii* Bolus (MP631) represented conflicts within the Afrotemperate clade. The remaining 17 phylogenetic conflicts were located within the Cape clade: *E.collina* Guthrie & Bolus (EO12613), *E.conferta* Andrews (MP887), *E.cruenta* Aiton (MP745 and MP999), *E.elimensis* L.Bolus (EO12843), *E.equisetifolia* Salisb. (ANA), *E.eugenea* Dulfer (EO12485), *E.fairii* Bolus (CM12), *E.grisbrookii* Guthrie & Bolus (EO12716), *E.intervallaris* Salisb. (MP556), *E.mollis* Andrews (CM5), *E.monadelphia* Andrews (FO2), *E.peziza* G.Lodd. [= *E.nivalis* Andrews] (MP719), *E.phillipsii* L.Bolus (MP1357), *E.recurvata* Andrews (EO12467), *E.rhopalantha* Dulfer (MP909), *E.stokoei* L.Bolus (MP825) and *E.turgida* Salisb. (S1962). After dividing these into separate cpDNA and nrDNA taxa, the combined supermatrix included 752 taxa. The resulting (multil-abelled) phylogenetic tree shows the same major geographically defined clades discovered in previous analyses, with newly-added accessions of Cape and Afrotemperate species consistently placed in Cape and TEA clades, respectively. The ML tree with summarised bootstrap support is presented in Suppl. material 7, along with both the single ML tree rate-smoothed under PL (represented in Fig. 1) and a sample of 100 rate-smoothed trees derived from bootstrap resampled data.

### ﻿EDGE analyses

Within *Erica*, 149 Ma of evolutionary history is at risk, of a total of 804 Ma (18%) represented by the genus. Thirty-nine species were identified as EDGE species (Table 1, Fig. 1) and 34 species are found on the EDGE Research list (Table 2).

**Table 1.** *Erica*EDGE Species: the list of 39 EDGE Species of *Erica* (ranked by median EDGE score). These are species which have an EDGE score above the median in at least 95% of the iterations (trees) and that are threatened. Note that DD/NE are excluded from this list. This follows the definition of EDGE Species in Gumbs et al. (2023). Clade: *Erica* clade to which a species is assigned (see text); Rank: overall EDGE rank; above.med: number of iterations in which the EDGE score of this species is above the median EDGE scores of all species; ED.med: median ED score from 100 trees; EDGE.med: median EDGE score from 100 trees; TBL.med: median terminal branch length from 100 trees; TBL%: percentage of ED attributed to the terminal branch length (rounded to the nearest decimal); RL.cat: IUCN Red List category.

Clade	Species	Overall EDGE rank	above.median_total	ED.med	EDGE.med	TBL.med	TBL%	RL.cat
EUR4	*E.maderensis* (Benth.) Bornm.	1	100	10.5439	9.8898	10.0860	95.7%	CR
TEA	*E.hillburttii* (E.G.H.Oliv.) E.G.H.Oliv.	14	99	1.4121	1.2389	1.1702	82.9%	CR
CAPE	*E.sagittata* Klotzsch ex Benth.	31	100	1.0621	0.5252	0.9217	86.8%	EN
TEA	*E.thomensis* (Henriq.) Dorr & E.G.H.Oliv.	36	98	0.4975	0.4214	0.3944	79.3%	CR
CAPE	*E.platycalyx* E.G.H.Oliv.	38	100	0.7239	0.3693	0.7209	99.6%	EN
CAPE	*E.pauciovulata* H.A.Baker	39	100	1.5021	0.3584	1.3918	92.7%	VU
CAPE	*E.vlokii* E.G.H.Oliv.	41	100	1.3418	0.3085	1.2818	95.5%	VU
CAPE	*E.cabernetea* E.G.H.Oliv.	45	99	0.2715	0.2536	0.1320	48.6%	CR
CAPE	*E.hermani* E.G.H.Oliv.	47	100	0.5207	0.2429	0.5088	97.7%	EN
CAPE	*E.juniperina* E.G.H.Oliv.	49	97	0.4950	0.2220	0.4901	99.0%	EN
CAPE	*E.extrusa* Compton	52	100	0.2396	0.2034	0.1284	53.6%	CR
CAPE	*E.oligantha* Guthrie & Bolus	56	100	0.3145	0.1584	0.2872	91.3%	EN
CAPE	*E.turgida* Salisb.	58	97	0.1680	0.1488	0.1640	97.6%	CR
CAPE	*E.ustulescens* Guthrie & Bolus	60	99	0.1621	0.1437	0.1504	92.8%	CR
TEA	*E.psittacina* E.G.H.Oliv. & I.M.Oliv.	61	95	0.2651	0.1354	0.2091	78.9%	EN
CAPE	*E.stylaris* Spreng.	62	99	0.5635	0.1346	0.5507	97.7%	VU
CAPE	*E.sociorum* L.Bolus	64	98	0.1417	0.1240	0.1339	94.5%	CR
CAPE	*E.jasminiflora* Salisb.	65	100	0.1349	0.1240	0.1349	100.0%	CR
CAPE	*E.karwyderi* E.G.H.Oliv.	66	97	0.1244	0.1226	0.1172	94.2%	CR
CAPE	*E.aneimena* Dulfer	69	98	0.4616	0.1121	0.4415	95.7%	VU
CAPE	*E.zebrensis* Compton	70	99	0.2545	0.1102	0.2360	92.7%	EN
CAPE	*E.gracilipes* Guthrie & Bolus	71	98	0.1185	0.1064	0.1182	99.7%	CR
CAPE	*E.zeyheriana* (Klotzsch) E.G.H.Oliv.	72	98	0.4618	0.1056	0.4579	99.2%	VU
CAPE	*E.perplexa* E.G.H.Oliv.	78	98	0.1079	0.0984	0.1079	100.0%	CR
CAPE	E.alexandrissp.acockii (Compton) E.G.H.Oliv. & I.M.Oliv.	82	99	0.0885	0.0885	0.0336	37.9%	EX
CAPE	E.alexandriGuthrie & Bolusssp.alexandri	83	99	0.0952	0.0885	0.0336	35.3%	CR
CAPE	E.bolusiaevar.cyathiformis H.A.Baker	86	97	0.0858	0.0832	0.0325	37.9%	CR
CAPE	*E.brachysepala* Guthrie & Bolus	87	97	0.1683	0.0809	0.1641	97.5%	EN
CAPE	E.bolusiaeT.M.Saltervar.bolusiae	89	96	0.0888	0.0795	0.0325	36.6%	CR
CAPE	*E.modesta* Salisb.	90	95	0.1446	0.0792	0.1366	94.5%	EN
CAPE	*E.tetrathecoides* Benth.	95	98	0.3047	0.0716	0.2899	95.1%	VU
CAPE	*E.garciae* E.G.H.Oliv.	97	98	0.2728	0.0711	0.2587	94.9%	VU
CAPE	*E.alfredii* Guthrie & Bolus	99	99	0.2807	0.0705	0.2712	96.6%	VU
CAPE	*E.hansfordii* E.G.H.Oliv.	101	96	0.0781	0.0690	0.0767	98.2%	CR
CAPE	*E.verticillata* P.J.Bergius	120	95	0.0591	0.0530	0.0504	85.2%	CR
CAPE	E.banksiassp.comptonii (T.M.Salter) E.G.H.Oliv. & I.M.Oliv.	125	97	0.1017	0.0506	0.0911	89.5%	EN
CAPE	*E.calcicola* (E.G.H.Oliv.) E.G.H.Oliv.	126	96	0.0981	0.0501	0.0979	99.8%	EN
CAPE	*E.multiflexuosa* E.G.H.Oliv.	127	95	0.2133	0.0500	0.1925	90.3%	VU
CAPE	E.filiformisSalisb.var.filiformis	163	97	0.1723	0.0388	0.1215	70.5%	VU

**Table 2.** EDGE Research List: the list of 34 species (ranked by median EDGE score) which have an EDGE score above the median, but which are of status data deficient or not evaluated (DD/NE). Gumbs et al. (2023) identify such species as part of the EDGE Research List. Column names as in Table 1.

Clade	Species	Overall EDGE rank	above.median_total	ED.med	EDGE.med	TBL.med	TBL%
EUR1	*E.spiculifolia* Salisb.	2	100	24.0947	5.2325	23.8520	99.0%
EUR2	E.siculassp.bocquetii (Peșmen) E.C.Nelson	3	100	21.3982	5.1615	17.1120	80.0%
EUR5	*E.australis* L.	4	100	17.6798	4.8616	17.6798	100.0%
EUR2	E.siculaGuss.ssp.sicula	5	100	21.7980	4.4735	17.1120	78.5%
EUR3	*E.umbellata* L.	6	100	15.8209	3.4070	15.4362	97.6%
EUR1	*E.carnea* L.	7	100	11.7892	2.7542	9.8654	83.7%
EUR1	*E.ciliaris* L.	8	100	14.7178	2.6840	14.6815	99.8%
EUR1	*E.erigena* R.Ross	9	100	10.8225	2.6388	9.8462	91.0%
EUR1	*E.terminalis* Salisb.	10	100	9.9058	2.5128	8.6242	87.1%
EUR1	*E.multiflora* L.	11	100	8.1951	2.1022	7.6223	93.0%
EUR1	*E.tetralix* L.	12	100	9.9433	1.7605	9.9058	99.6%
EUR1	*E.numidica* (Maire) Romo & Borat.	13	100	6.1181	1.3575	4.6816	76.5%
EUR1	*E.manipuliflora* Salisb.	16	100	3.9974	0.9240	3.9458	98.7%
KIN	E.kingaensisssp.bequaertii (De Wild.) R.Ross	17	97	2.7247	0.7081	1.8967	69.6%
TEA	E.caffrorumvar.luxurians Bolus	19	98	2.4957	0.6172	1.8676	74.8%
EUR1	*E.platycodon* (Webb & Berthel.) Rivas Mart., Capelo, J.C.Costa, Lousã, Fontinha, R.Jardim & M.Seq. ssp.platycodon	20	98	2.3410	0.6104	1.5741	67.2%
TRIM	E.trimerassp.meruensis (R.Ross) Dorr	21	99	2.3811	0.6100	1.9316	81.1%
KIN	E.kingaensisEngl.ssp.kingaensis	22	95	2.7433	0.6032	2.0653	75.3%
TRIM	E.trimerassp.keniensis (S.Moore) Beentje	23	100	2.6154	0.5944	2.2616	86.5%
TRIM	E.trimerassp.kilimanjarica (Hedberg) Beentje	25	99	2.3724	0.5911	1.6678	70.3%
TRIM	E.trimerassp.abyssinica (Pic.Serm. & Heiniger) Dorr	26	98	2.4190	0.5707	1.7166	71.0%
TRIM	E.trimera(Engl.)Beentjessp.trimera	27	100	2.3415	0.5696	2.0926	89.4%
EUR1	*E.scoparia* L.	28	100	2.0276	0.5475	1.6054	79.2%
EUR1	E.platycodonssp.maderincola (D.C.McClint.) Rivas Mart., Capelo, J.C.Costa, Lousã, Fontinha, R.Jardim & M.Seq.	29	98	2.5841	0.5337	1.9248	74.5%
TEA	*E.drakensbergensis* Guthrie & Bolus	30	96	1.8822	0.5262	1.3220	70.2%
TEA	*E.caffrorumBolusvar.caffrorum	32	97	2.7462	0.4863	1.8676	68.0%
EUR1	*E.azorica* Hochst. ex Seub.	33	98	2.0295	0.4502	1.5852	78.1%
TEA	*E.mauritiensis* E.G.H.Oliv.	34	98	1.9370	0.4382	1.8783	97.0%
TRIM	E.trimerassp.elgonensis (Mildbr.) Beentje	35	96	2.1595	0.4377	1.8427	85.3%
TEA	*E.whyteana* Britten	37	95	1.9573	0.3727	1.7905	91.5%
TEA	*E.microdonta* (C.H.Wright) E.G.H.Oliv.	48	95	1.3526	0.2370	1.2561	92.9%
TEA	*E.galioides* Lam.	50	95	1.0123	0.2097	0.7480	73.9%
CAPE	*E.orientalis* R.A.Dyer	74	96	0.2856	0.1024	0.2723	95.3%
CAPE	*E.gibbosa* Klotzsch ex Benth.	79	95	0.4205	0.0981	0.4139	98.4%

*Threat status for Ericacaffrorumssp.caffrorum was mistakenly omitted: it has been assigned the LC category and, therefore, can be disregarded as a member of the Research list.

### ﻿Priority areas

**Figure 2.**
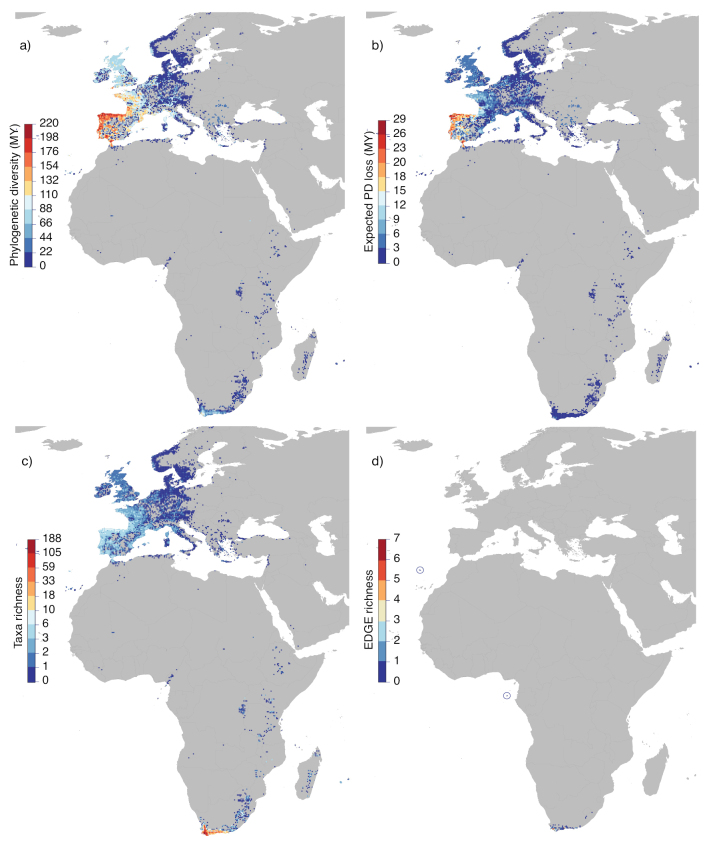
Global distribution of *Erica*: **a** phylogenetic diversity (PD; in millions of year, MY) **b** expected PD loss (in millions of year, MY) **c** taxon richness; and **d**EDGE species richness. Note: the only EDGE species found outside of South Africa are *E.maderensis* from Madeira and *E.thomensis* from São Tomé and Príncipe; these islands are circled in map **d**) (upper left and centre, respectively).

**Figure 3.**
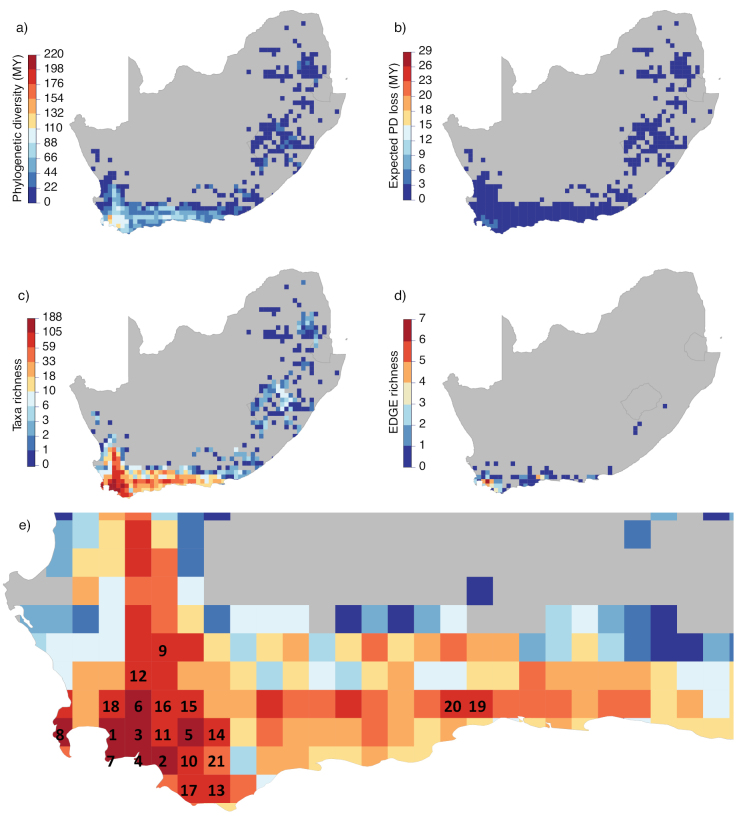
South African distribution of *Erica***a** phylogenetic Diversity (PD) **b** expected PD loss **c** taxon richness; and **d**EDGE species richness. The scales follow those presented in Fig. 2 at the global level (i.e. from zero to the global maximum). In **e** the highest scoring Quarter Degree Squares (QDS) for taxon richness in the region are numbered following Table 3 (colour coding as per c).

Mapping of taxon richness per QDS illustrates the disparity between the Cape Floristic Region and all other areas of the distribution (Fig. 2c), with taxon richness of 100 or more in 13 QDS between 33–34°S and 18–19°E in the Western Cape (Table 3). Geographical patterns of phylogenetic diversity (PD) and expected PD loss (i.e. the amount of evolutionary history expected to be lost give extinction of taxa) are similar to each other and highest overall around the Atlantic coast of the Iberian Peninsula, whilst, in the Southern Hemisphere, they are highest in the Cape within the region of top taxon diversity. Cape PD peaks in the ‘Stellenbosch’ QDS, followed by ‘Somerset West’ and ‘Stanford’ (Table 3; Fig. 3a). Within the Cape Region, there is overlap between area PD and EDGE taxon richness (both high in "Somerset West"; Fig. 3), but no obvious link: the QDS with high EDGE taxon richness correspond to different QDS within the Overberg region ("Grabouw", followed by "Hermanus" and "Caledon") with a more distant regional peak ("Jonkersberg") in the eastern Langeberg. "Stellenbosch", with highest PD and taxon richness, scores lowest in terms of EDGE taxon richness (Table 3).

**Table 3.** Southern Hemisphere QDS that scored highest for taxon richness (≥ 100), PD (≥ 90), EDGE taxon richness (≥ 3) and expected PD loss, sorted by taxon richness. All are in the Western Cape; they are indicated by numbers in Fig. 3e. The QDS that scored highest overall for PD, in Galicia, northern Spain, is included for comparison. Numbers in bold indicate the highest value for each metric.

Fig. 3e	Name	QDS	X	Y	PD.med	ePDloss.med	Taxon richness	Edge richness
1	Somerset West	3418BB	18.875	-34.125	117.45	5.23	**188**	5
2	Stanford	3419AD	19.375	-34.375	111.80	4.96	162	4
3	Grabouw	3419AA	19.125	-34.125	105.29	4.27	150	**7**
4	Hermanus	3419AC	19.125	-34.375	105.35	4.46	139	5
5	Greyton	3419BA	19.625	-34.125	101.18	3.24	130	2
6	Franschhoek	3319CC	19.125	-33.875	102.44	2.69	127	3
7	Hangklip	3418BD	18.875	-34.375	100.17	2.92	126	3
8	Cape Peninsula	3418AB	18.375	-34.125	96.69	2.72	113	3
9	Ceres	3319AD	19.375	-33.375	96.31	1.39	111	0
10	Jongensklip	3419BC	19.625	-34.375	96.37	3.50	110	3
11	Caledon	3419AB	19.375	-34.125	95.17	2.59	103	5
12	Bain’s Kloof	3319CA	19.125	-33.625	86.06	1.78	100	2
13	Elim	3419DB	19.875	-34.625	84.92	2.26	100	3
14	Riviersonderend	3419BB	19.875	-34.125	90.90	2.44	97	2
15	Langvlei	3319DC	19.625	-33.875	90.05	1.62	96	1
16	Villiersdorp	3319CD	19.375	-33.875	96.33	2.19	95	1
17	Baardskeerdersbos	3419DA	19.625	-34.625	75.88	2.05	86	4
18	Stellenbosch	3318DD	18.875	-33.875	**143.43**	5.36	82	1
19	George	3322CD	22.375	-33.875	96.87	2.32	80	4
20	Jonkersberg	3322CC	22.125	-33.875	94.99	2.43	77	5
21	Napier	3419BD	19.875	-34.375	66.13	1.92	62	3
-	Galicia, Spain	-	-7.875	43.125	**219.96**	**29.19**	11	0

## ﻿Discussion

### ﻿Inverted patterns of taxon richness and phylogenetic diversity in *Erica*

Summarising taxon richness, phylogenetic diversity and EDGE taxon richness reveals stark contrasts across the distribution of *Erica*. Whilst Cape *Erica* species greatly outnumber those from other regions, the Cape clade is no older than the other African *Erica* clades and considerably younger than the European ones (Pirie et al. 2016): the species are, on average, much more closely related, individually representing less unique phylogenetic diversity. In plants, in general, local species radiations contribute to regional disparities in species richness that tend to be greater than the corresponding differences in PD (Tietje et al. 2023). The rapid radiation in Cape *Erica* (Pirie et al. 2016) results in an inversion of the disparity at the QDS level: taxon richness is lowest and PD highest in Europe, whilst the by far highest taxon richness found in the Cape (Oliver et al. 1983) is only reflected in moderate to low PD (Fig. 2).

Oliver et al. (1983) analysed patterns of taxon richness across the whole Cape flora. As the largest genus in the CFR, *Erica* data contributed significantly to the results of the Oliver et al. (1983) analysis. It is, therefore, perhaps unsurprising that the QDS that they identified as having the highest taxon richness, ‘Somerset West’ (3418BB; which includes a fynbos-rich mountainous region straddling both the northern part of the Kogelberg Biosphere Reserve and southern end of the Hottentots Holland Nature Reserve), also harbours the highest taxon richness of *Erica* in data analysed here. Taxon richness decreases towards the east and north from this peak in the south-western corner of the Western Cape, both for *Erica* and for plants in general (Levyns 1964; Goldblatt 1978; Linder 2003; Forest et al. 2007; Colville et al. 2020), a pattern that was referred to as “Levyns’ Law” by Cowling et al. (2017). Explanations for the causes of high species richness in the SW Cape include lower extinction rates of seeder lineages concentrated in this area of winter rainfall (Cowling et al. 2018). The highest *Erica*PD in the Cape is found in ‘Stellenbosch’ (3318DD), adjacent to ‘Somerset West’ and PD roughly tracks taxon richness regionally. The epicentre of *Erica*PD in the Cape is, therefore, found within a much smaller total area than European peak PD around the Atlantic coast of the Iberian Peninsula, where it is represented by a relatively homogenous suite of distantly related, mostly widespread taxa. For comparisons of PD and associated metrics within regions such as the Cape, it may be important to take into account the impact of such disparities between regions. For example, a similar analysis within the Cape clade only would most likely reveal EDGE species from this region that are not identified in the global analysis.

The relationship between taxon richness, PD and EDGE taxon richness is not direct: the richest areas do not necessarily include much threatened PD. This is abundantly clear when comparing Europe to other areas, but also the case when comparing within the Cape. Although the highest EDGE score in the Cape, in ‘Grabouw’ (3419AA), is also in the hyper-diverse south-west, we identified one area further east that also shows amongst the highest values for EDGE taxon richness (‘Jonkersberg’, 3322CC). Individual *Erica* taxa are often narrowly endemic within the Cape, resulting in a rapid geographic turnover of species assemblages. Since threat status of taxa is in part dependent on the conservation status of habitats (threatened taxa tend to be local endemics that are not in protected areas), high regional EDGE scores may reflect a local shortfall in coverage of endemic taxa by protected areas and, hence, point to a need for conservation action outside the most obviously diverse regions.

### ﻿Threat assessments and alpha taxonomy needed to identify more EDGE species

Despite its lower overall PD, South Africa’s Cape clade still comprises most of the *Erica* taxa identified as EDGE species. Of 1048 *Erica* taxa, we identified 39 EDGE species, i.e. taxa known to be threatened and scoring above median EDGE values for the genus in 95% or more of the iterations (i.e. trees). All but four are members of the Cape clade. The only EDGE species found outside of the Cape Region are the critically endangered *E.maderensis* (Benth.) Bornm. found only on Madeira, *E.thomensis* (Henriq.) Dorr & E.G.H.Oliv. endemic to São Tomé and Príncipe and *E.hillburttii* (E.G.H.Oliv.) E.G.H.Oliv. from the north-eastern Eastern Cape and *E.psittacina* E.G.H.Oliv. & I.M.Oliv. found in adjacent KwaZulu-Natal.

Several gaps in fundamental knowledge can be assumed to have depressed both the number of *Erica*EDGE species and regional EDGE taxon richness values, particularly with regard to wider African and Madagascan species diversity. A particular challenge is the lack of threat assessments for 274 taxa within *Erica*.

Worldwide, both Madagascar and South Africa have amongst the highest numbers of species that are unassessed, but predicted to be threatened (Bachman et al. 2023). In South Africa, the proportion of taxa that have been assessed is high (87%) compared with other regions of high endemism such as Mexico or Brazil (24% and 28%, respectively; Gallagher (2023)). In total, 190 of 944 South African *Erica* taxa are known to be threatened (VU, EN or CR; Raimondo et al. (2009)). This is lower than the global figure of 39% cited by Nic Lughadha et al. (2020), but the *Erica* numbers do not include the over 100 taxa classified as ‘rare’ by SANBI, nor, importantly, the further 100 plus assessed as Data Deficient (DD) or Not Evaluated (NE). Many of these are also likely to be rare. Bachman et al. (2023) estimated that 69% of DD species are likely to be threatened, of which 86% with high certainty.

This important knowledge gap is reflected in the EDGE research list, comprising taxa that have an EDGE score above the median in more than 95% of trees, but that are either DD or NE. This list includes a very different suite of taxa, predominantly representatives of the minority, non-Cape clades. Not all of these are of immediate concern: the widespread European species of *Erica*, while not formally assessed, are unlikely to be threatened. However, there are narrowly distributed species, such as the endemic Iberian *E.andevalensis* Cabezudo & J.Rivera and *E.mackayana* Bab. (Rodríguez-Buján et al. 2024), which, as close sister species, are mutually excluded from either EDGE or research lists, but may nevertheless be of concern. Those with restricted island and coastal Mediterranean distributions, such as taxa of the wind pollinated *E.scoparia* L. / *E.platycodon* (Webb & Berthel.) Rivas Mart., Capelo, J.C.Costa, Lousã, Fontinha, R.Jardim & M.Seq. complex and *E.sicula* complex, require assessment (Pasta et al. 2024). There is also regional variation, such as represented by *Ericanumidica* (Maire) Romo & Borat. (Romo & Boratynski, 2010) which is currently included within the widespread *Ericacinerea* L. (Nelson, 2011), but would otherwise be considered threatened in its restricted range in Algeria (Hamel et al. 2021).

Formal assessments – even of common species – would be useful to confirm their status. Although the threat status of a substantial proportion of South African species has been assessed (including over 80% of Cape clade taxa), current figures were not updated within the last decade (Raimondo et al. 2009). In other regions across Africa and Madagascar with lower species richness, but generally higher phylogenetic diversity per species, there have been far fewer threat assessments (less than 25% of taxa outside the Cape clade).

Clearly, neither the EDGE List nor the EDGE research list can include undescribed species diversity. For Africa, Ondo et al. (2023) estimated that the greatest shortfall in plant species remaining to be described and geolocated were in Madagascar and Cape Provinces - i.e. centres of *Erica* diversity - and that species with small geographic ranges were more likely to remain undescribed. The shortfall for the poorly-understood Madagascan taxa is known (Dorr, in prep.) and even the better-known South African flora includes numerous putative undescribed species, often local endemics (Hoekstra et al., in prep.), as well as diversity within species complexes potentially under-represented by formal taxa (Pirie et al. 2017; Musker et al. 2023). These also lack formal threat status and are not taken into account in our overviews of diversity and endemism. Such undescribed and range-restricted species are more likely to be threatened (Brown et al. 2023).

### ﻿Improving the phylogenetic hypothesis for *Erica*

The phylogenetic hypothesis presented here represents a further improvement on previous work (McGuire and Kron 2005; Pirie et al. 2011, 2016; Mugrabi de Kuppler et al. 2015), including more species, improved resolution and one further nrDNA sequence marker to validate results based on ITS. The phylogenetic tree has already been used for the inference of ancestral wood anatomy within *Erica* (Akinlabi et al. 2023) and as a means to control for phylogenetic signal in analyses of the impact of flower colour on nectar robbing (Coetzee et al., in prep.). It will also be an important tool for identifying and testing the closest relatives of undescribed species diversity (Hoekstra et al., in prep.). However, there is still a substantial shortfall in representation of species and their genomes and in phylogenetic resolution.

Despite our clade-based inclusion of taxa not being represented in the phylogenetic tree, in almost all cases, these will fail to feature on EDGE lists until their precise relationships are known. The subspecies of *E.trimera* and of *E.kingaensis* are exceptions, featuring on the EDGE research list due to the isolated positions of these species in the African *Erica* clade. The *E.trimera* subspecies are closely related according to the results of Gizaw et al. (2013), but we are unable to confirm this for the subspecies of *E.kingaensis* due to the lack of equivalent data. All the subspecific taxa of a species were grouped together by the imputation approach if they were not already included in the phylogenetic tree. Even where we have DNA sequence data, the remaining (and considerable) phylogenetic uncertainty within the Cape clade will serve to average out the diversity of individual taxa where they are not placed with confidence and will, therefore, also likely depress the number of EDGE species.

Given these factors, the current EDGE list for *Erica* must be viewed as a conservative underestimate, to aid focusing research and conservation priorities, but not to the exclusion of action where data are incomplete.

### ﻿Future research

Successful targeting and implementation of conservation efforts, both in-situ and ex-situ, require improved understanding of taxonomy, species boundaries, distributions, genetic diversity, morphology, ecology and threat levels. By providing the current phylogenetic resources (e.g. data, protocols, Musker et al., in prep.) and tools to aid effective identification of species (Oliver et al. 2024), we can improve both phylogenetic and alpha taxonomic knowledge. Gathering sequence data for putative undescribed or cryptic diversity (of species or subspecific taxa) may help identify closest relatives and focus diagnoses (Hoekstra et al., in prep.) or even assist in complex species delimitation challenges, particularly with high-throughput DNA sequencing approaches (Musker et al. 2023).

Updated and new threat assessments are needed and these results may help in prioritising work given limited resources. A potential route forward could be to use automated preliminary assessments to target DD and NE species that are likely to be threatened, whilst deprioritising those that can be assumed with confidence to be of least concern (Bachman et al. 2023). Such assessments are dependent on the available distribution data, which, given the concentration of PD in regions close to the City of Cape Town, would be important to audit for potential sampling bias and to target fieldwork.

Trends in habitat and population persistence are an important aspect of threat assessments. Areas subject to formal protection may be spared direct human-mediated habitat destruction, but will not necessarily be resilient to impact of invasive species, changes to the fire regime or climate change. Predictions for the Cape indicate both warming and decline in winter rainfall, with Lötter & Le Maitre (2014) predicting long term species extinctions of 23% in the fynbos biome. The likely impact, for example on high mountain versus lowland species of *Erica*, is still largely unclear. Analysing the genus *Thesium* in the CFR, Zhigila et al. (2023) used niche modelling to project past, current and future distributions and tested for phylogenetic signal in range size, niche specialisation and threat status. They concluded that species at greatest risk were not more closely related than might be expected by chance and that the range of some species would decrease whilst others increased under projected climatic conditions. This would seem to support conservation prioritisation based on EDGE in addition to a case-by-case assessment of the future prospects for individual species. Equivalent work would be highly valuable, despite the greater scale of the task, with the numerous species of *Erica*.

## ﻿Conclusions

With an improved phylogenetic hypothesis and existing threat status assessments, we have identified 39 evolutionarily distinct and globally endangered (EDGE) taxa out of the over 1,000 currently recognised in the megagenus *Erica*. All but two EDGE taxa are from South Africa and all but four are endemic to the Cape Floristic Region. Using openly accessible distribution data, we were able to map taxon and phylogenetic diversity as well as EDGE taxon richness to regions of the *Erica* distribution. The results serve to highlight both particular threatened taxa and areas beyond the known centres of diversity and endemism as priorities for further research and conservation action. As widely recognised, such analyses are qualified by the grave limitations of our basic knowledge (Pollock et al. 2020). Ours represents a conservative underestimate of threatened *Erica*PD: an additional EDGE research list includes 34 evolutionarily distinct taxa for which threat status is unknown and substantial numbers of yet unsampled (and undescribed) taxa do not feature at all. This work will aid prioritisation of future research and conservation action, feeding directly into action through the Global Conservation Consortium for *Erica* (Pirie et al. 2022).

## ﻿Additional information

### Author ORCIDs

Michael D. Pirie https://orcid.org/0000-0003-0403-4470

Dirk U. Bellstedt https://orcid.org/0000-0002-6376-4855

Roderick W. Bouman https://orcid.org/0000-0002-2949-3318

Jaime Fagúndez https://orcid.org/0000-0001-6605-7278

Berit Gehrke https://orcid.org/0000-0001-5866-4430

Martha Kandziora https://orcid.org/0000-0002-1197-6207

Nicholas C. Le Maitre https://orcid.org/0000-0001-5122-3026

Seth Musker https://orcid.org/0000-0002-1456-1373

Ethan Newman https://orcid.org/0000-0002-9678-4895

Nicolai M. Nürk https://orcid.org/0000-0002-0471-644X

Sebastian Pipins https://orcid.org/0000-0001-9619-6957

Timotheus van der Niet https://orcid.org/0000-0002-5250-8995

Félix Forest https://orcid.org/0000-0002-2004-433X

### Data availability

All of the data that support the findings of this study are available in the main text or Supplementary Information.

## Supplementary materials

Supplementary material 1 Extended list of 1048 *Erica* species, subspecies and varieties used in the EDGE analyses Data type xlsx https://binary.pensoft.net/file/1088574This dataset is made available under the Open Database License (http://opendatacommons.org/licenses/odbl/1.0/). The Open Database License (ODbL) is a license agreement intended to allow users to freely share, modify, and use this Dataset while maintaining this same freedom for others, provided that the original source and author(s) are credited.Michael D. Pirie, Dirk U. Bellstedt, Roderick W. Bouman, Jaime Fagúndez, Berit Gehrke, Martha Kandziora, Nicholas C. Le Maitre, Seth Musker, Ethan Newman, Nicolai M. Nürk, E. G. H. Oliver, Sebastian Pipins, Timotheus van der Niet, Félix Forest

Supplementary material 2 Accessions table Data type xlsx https://binary.pensoft.net/file/1088575This dataset is made available under the Open Database License (http://opendatacommons.org/licenses/odbl/1.0/). The Open Database License (ODbL) is a license agreement intended to allow users to freely share, modify, and use this Dataset while maintaining this same freedom for others, provided that the original source and author(s) are credited.Michael D. Pirie, Dirk U. Bellstedt, Roderick W. Bouman, Jaime Fagúndez, Berit Gehrke, Martha Kandziora, Nicholas C. Le Maitre, Seth Musker, Ethan Newman, Nicolai M. Nürk, E. G. H. Oliver, Sebastian Pipins, Timotheus van der Niet, Félix Forest

Supplementary material 3 Google map of accessions Data type kmz https://binary.pensoft.net/file/1088576This dataset is made available under the Open Database License (http://opendatacommons.org/licenses/odbl/1.0/). The Open Database License (ODbL) is a license agreement intended to allow users to freely share, modify, and use this Dataset while maintaining this same freedom for others, provided that the original source and author(s) are credited.Michael D. Pirie, Dirk U. Bellstedt, Roderick W. Bouman, Jaime Fagúndez, Berit Gehrke, Martha Kandziora, Nicholas C. Le Maitre, Seth Musker, Ethan Newman, Nicolai M. Nürk, E. G. H. Oliver, Sebastian Pipins, Timotheus van der Niet, Félix Forest

Supplementary material 4 Synonymy table used for parsing GBIF data Data type xlsx https://binary.pensoft.net/file/1088577This dataset is made available under the Open Database License (http://opendatacommons.org/licenses/odbl/1.0/). The Open Database License (ODbL) is a license agreement intended to allow users to freely share, modify, and use this Dataset while maintaining this same freedom for others, provided that the original source and author(s) are credited.Michael D. Pirie, Dirk U. Bellstedt, Roderick W. Bouman, Jaime Fagúndez, Berit Gehrke, Martha Kandziora, Nicholas C. Le Maitre, Seth Musker, Ethan Newman, Nicolai M. Nürk, E. G. H. Oliver, Sebastian Pipins, Timotheus van der Niet, Félix Forest

Supplementary material 5 Presence/absence of taxa per QDS and numbers of QDS per taxon Data type zip https://binary.pensoft.net/file/1088578This dataset is made available under the Open Database License (http://opendatacommons.org/licenses/odbl/1.0/). The Open Database License (ODbL) is a license agreement intended to allow users to freely share, modify, and use this Dataset while maintaining this same freedom for others, provided that the original source and author(s) are credited.Michael D. Pirie, Dirk U. Bellstedt, Roderick W. Bouman, Jaime Fagúndez, Berit Gehrke, Martha Kandziora, Nicholas C. Le Maitre, Seth Musker, Ethan Newman, Nicolai M. Nürk, E. G. H. Oliver, Sebastian Pipins, Timotheus van der Niet, Félix Forest

Supplementary material 6 DNA sequence alignments Data type zip https://binary.pensoft.net/file/1088579This dataset is made available under the Open Database License (http://opendatacommons.org/licenses/odbl/1.0/). The Open Database License (ODbL) is a license agreement intended to allow users to freely share, modify, and use this Dataset while maintaining this same freedom for others, provided that the original source and author(s) are credited.Michael D. Pirie, Dirk U. Bellstedt, Roderick W. Bouman, Jaime Fagúndez, Berit Gehrke, Martha Kandziora, Nicholas C. Le Maitre, Seth Musker, Ethan Newman, Nicolai M. Nürk, E. G. H. Oliver, Sebastian Pipins, Timotheus van der Niet, Félix Forest

Supplementary material 7 Phylogenetic trees (cpDNA, nrDNA, combined) Data type zip https://binary.pensoft.net/file/1088580This dataset is made available under the Open Database License (http://opendatacommons.org/licenses/odbl/1.0/). The Open Database License (ODbL) is a license agreement intended to allow users to freely share, modify, and use this Dataset while maintaining this same freedom for others, provided that the original source and author(s) are credited.Michael D. Pirie, Dirk U. Bellstedt, Roderick W. Bouman, Jaime Fagúndez, Berit Gehrke, Martha Kandziora, Nicholas C. Le Maitre, Seth Musker, Ethan Newman, Nicolai M. Nürk, E. G. H. Oliver, Sebastian Pipins, Timotheus van der Niet, Félix Forest
