# Supplementary figures and images for: ﻿Spatial decoupling of taxon richness, phylogenetic diversity and threat status in the megagenus Erica (Ericaceae)
Source: PhytoKeys. 2024 Jul 10;244:127–50. doi: 10.3897/phytokeys.244.124565 (PMC11255470; doi:10.3897/phytokeys.244.124565)

Cape

Tropical East Africa

Europe

Outgroup

0.03

bootstrap support

0

70

100

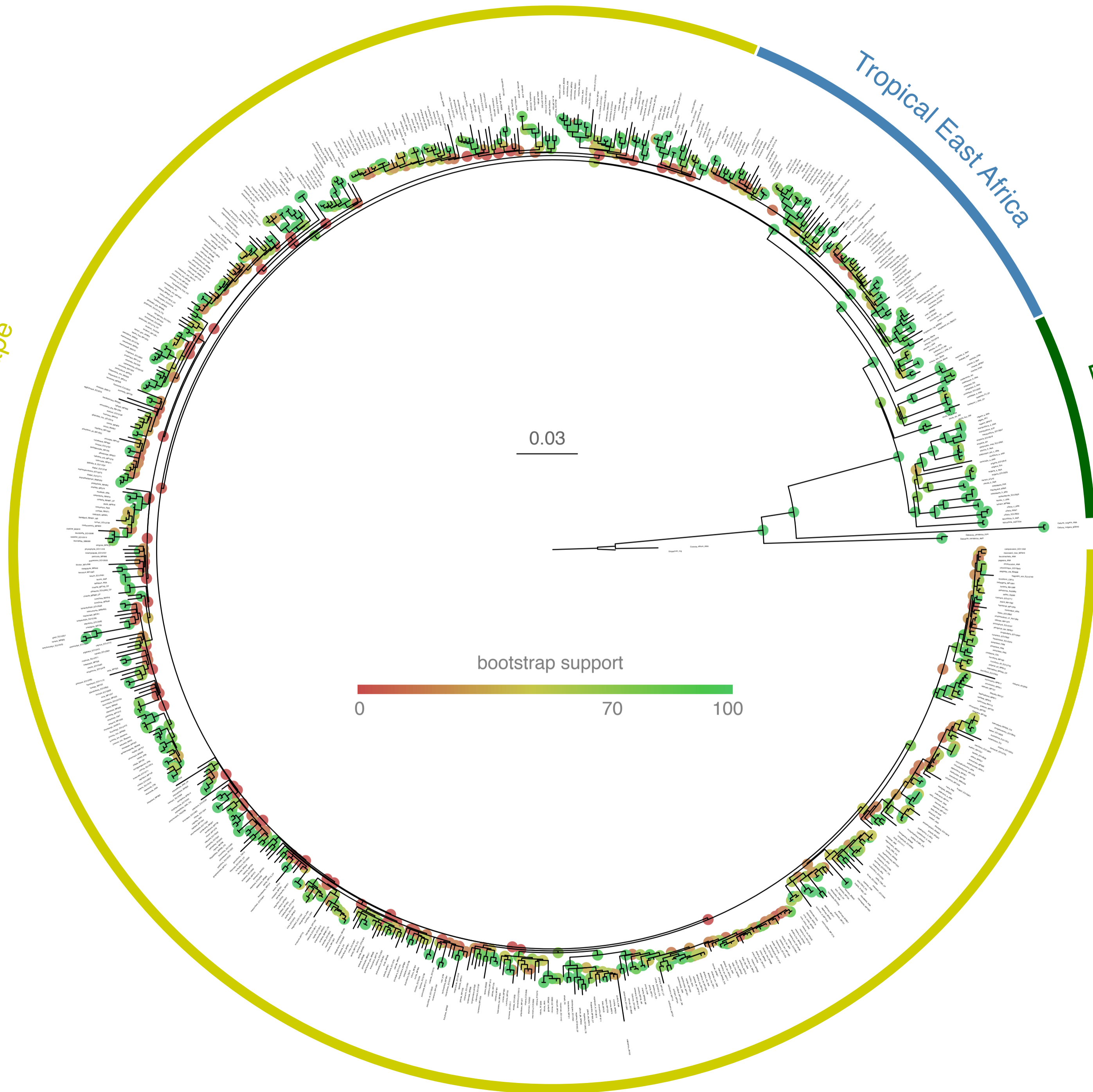

Supplement: Supplementary material 7 — Phylogenetic trees (cpDNA, nrDNA, combined) [file phytokeys-244-127_article-124565__-s007.zip › Appendix 7 trees/Fig_S1_MLtre(1).pdf]

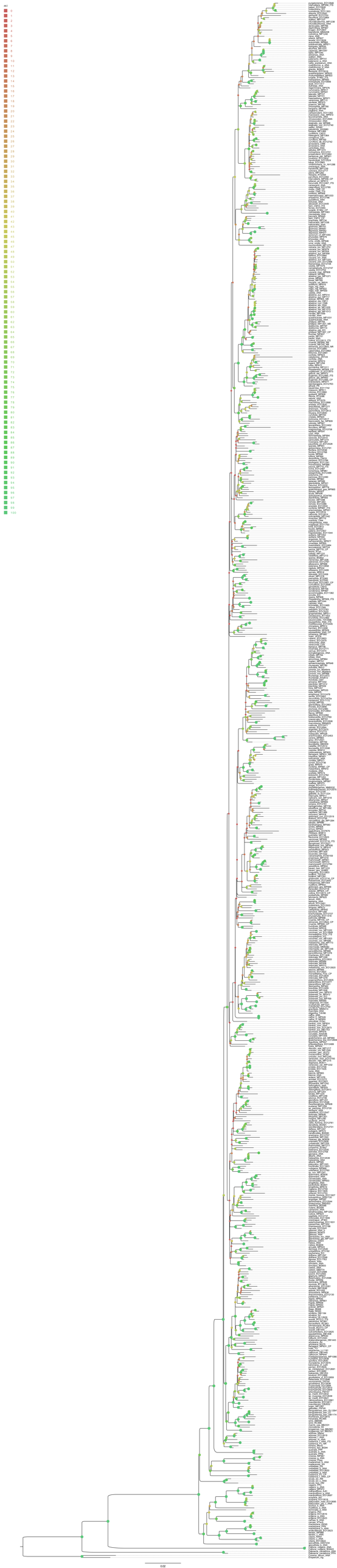

Supplement: Supplementary material 7 — Phylogenetic trees (cpDNA, nrDNA, combined) [file phytokeys-244-127_article-124565__-s007.zip › Appendix 7 trees/RAxML_bipartitions.Erica_supermatrix_20220701_taxa752_ed.tre.pdf]
